# Sensing Uranyl(VI) Ions by Coordination and Energy Transfer to a Luminescent Europium(III) Complex

**DOI:** 10.1002/anie.201805316

**Published:** 2018-06-29

**Authors:** Peter Harvey, Aline Nonat, Carlos Platas‐Iglesias, Louise S. Natrajan, Loïc J. Charbonnière

**Affiliations:** ^1^ The Centre for Radiochemistry Research School of Chemistry, The University of Manchester Brunswick Street Manchester M13 9PL UK; ^2^ Laboratoire d'Ingénierie Moléculaire Appliquée à l'Analyse, IPHC, UMR 7178 ECPM 25 rue Becquerel 67087 Strasbourg Cedex 02 France; ^3^ Centro de Investigaciones Científicas Avanzadas (CICA), and Departamento de Química Universidade da Coruña Campus da Zapateira-Rúa da Fraga 10 15008 A Coruña Spain

**Keywords:** energy transfer, europium, luminescence, sensors, uranyl

## Abstract

The release of uranyl(VI) is a hazardous environmental issue, with limited ways to monitor accumulation in situ. Here, we present a method for the detection of uranyl(VI) ions through the utilization of a unique fluorescence energy transfer process to europium(III). Our system displays the first example of a “turn‐on” europium(III) emission process with a small, water‐soluble lanthanide complex triggered by uranyl(VI) ions.

The development of nuclear technologies has led to many cases of accidental and intentional release of radionuclides, with accumulation of significant levels of uranium in the environment.^1^ Of particular concern is the uranyl(VI) cation, UO_2_^2+^. This species, a potent nephrotoxin,^2^ is highly mobile in groundwater and biological systems, leading to possible problematic spread of radiotoxic material following containment breaches.

To date, there has been limited development of probes for UO_2_^2+^ detection, with scintillation counting and X‐ray based methods generally preferred.1a While these allow determination of total uranium content they, importantly, cannot distinguish between different oxidation states and, compared to fluorescence‐based techniques, are limited in their in situ application. This limitation hinders the real‐time and remote monitoring of remediation strategies, such as the biotic reduction of UO_2_^2+^ to more immobile U^IV^‐containing minerals, a strategy currently under development as a bioremediation tool.1a The few luminescence‐based detection systems reported to date^3^ have failed to exploit the intrinsic photophysical properties of UO_2_^2+^, which allow distinct identification over other oxidation states and, with the correct design, afford an opportune and selective handle with which to monitor local concentration fluctuations of this environmentally hazardous species.

The intrinsic photophysical properties of the UO_2_^2+^ cation arise from formally forbidden charge transfer transitions from oxo‐based molecular orbitals to nonbonding, unoccupied f‐orbitals.^4^ While direct interpretation of these transitions can be complicated by speciation and spectral overlap with optical transitions from biological media,^5^ they do provide a means for indirect UO_2_^2+^ detection via energy transfer to other longer wavelength (and longer‐lived) emissive species. Of particular interest here is the spectral overlap of the UO_2_^2+^ emission (ca. 520 nm) and the europium(III) excitation bands (principally ^5^D_1_←^7^F_0,1_),^6^ which enable efficient energy transfer to occur from the former to the latter (Scheme 1).

**Scheme 1:**
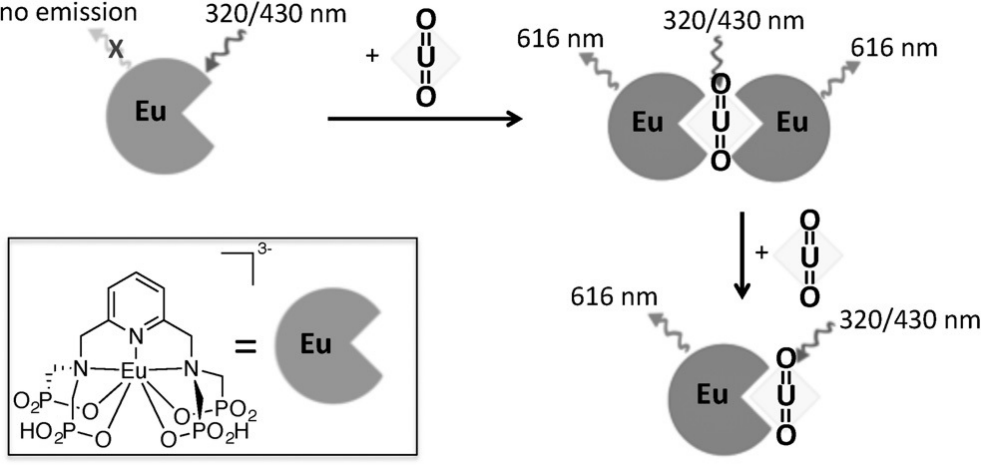
“Turn‐on” emission of [EuL] at selected excitation wavelengths due to energy transfer from UO_2_^2+^.

Here, we report the first example of UO_2_^2+^ to lanthanide energy transfer in a water‐soluble, molecular europium(III) complex, [EuL].^7^ We suggest that this energy transfer could provide a highly selective method of UO_2_^2+^ detection, due to the unique photophysical properties of UO_2_^2+^ that allow this process to occur.

Initial spectrophotometric titrations were performed by following the absorption, steady‐state emission and excitation spectra of [EuL] as a function of added UO_2_^2+^ (Figures 1, S1 and S2). In the absence of UO_2_^2+^, the emission spectrum of [EuL] upon ligand excitation (280 nm) is typical of Eu^3+^ emission with the narrow emission bands corresponding to the ^5^D_0_→^7^F_*J*_ transitions (578, 595, 613, 654 and 702 nm for *J*=0 to 4, respectively).^7^, ^8^ Addition of uranyl(VI) nitrate (0–2 equivalents) at pH 7.4 led to a decrease of the overall Eu^3+^ emission intensity observed upon ligand‐centered excitation at 280 nm (Figure 1). No significant changes are seen in the emission pattern or in the ^5^D_0_→^7^F_0_ transition, pointing to minor variations in the coordination sphere of the Eu^3+^ species under these conditions (Table S4 and Figure S8). The decrease in ligand‐excitation efficiency can be explained by the strong competing absorption associated with the increasing presence of UO_2_^2+^ species (Figure S1).

**Figure 1:**
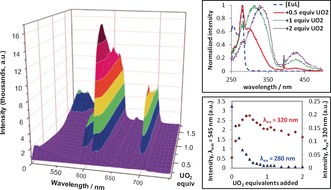
Left: Emission spectra of [EuL] upon addition of UO_2_^2+^ nitrate ([EuL]=8.2×10^−4^ m, 0.01 m TRIS buffer, pH 7.4, *λ*_exc_=320 nm). Note initial emission around 500 nm is ligand‐based fluorescence. Top right: Normalized excitation spectra (*λ*_em_=613 nm) of [EuL] upon addition of UO_2_^2+^, indicating growth of distinct UO_2_^2+^ bands at 320/430 nm. Bottom right: Intensity of Eu^3+^ emission at 613 nm upon addition of UO_2_^2+^ with excitation into the ligand‐centred (280 nm, blue) and the UO_2_^2+‐^centred (320 nm, red) bands.

The appearance of two new excitation bands (*λ*_em_=613 nm) at 430 and 320 nm was observed upon addition of UO_2_^2+^ (Figures 1 and S2). Such bands are characteristic of the presence of UO_2_^2+^ complexes in solution and could only realistically be attributed to UO_2_^2+^ transitions from the Laporte forbidden O‐to‐U ligand‐to‐metal charge transfer (LMCT) transition and from the LMCT from the equatorial ligands, respectively.^9^ The excitation spectra clearly suggest that the energy absorbed by UO_2_^2+^, or by its corresponding hydrolysis species, is transferred to Eu^3+^. Moreover, a red shift (ca. 14 nm) in the band at 330 nm is observed upon further addition of uranyl(VI) nitrate, providing evidence for the alteration of the equatorial coordination environment of the UO_2_^2+^ ion from 0.5 to 1 equivalents. Such energy transfer from UO_2_^2+^, and other actinides, to the ^5^D_0_ excited state of Eu^3+^ is known to be efficient in solid matrixes, polymers and glasses;^10^ however, it has previously only been seen in aqueous solution with highly concentrated mixtures.^11^

Further titrations were carried out upon excitation into the uranyl‐based LMCT transitions at 320 nm (Figure 1) and 420 nm (Figure S3). As expected, exciting into the UO_2_^2+^ LMCT bands led to Eu^3+^ emission from the ^5^D_0_ excited state. Significant variations are observed in the emission intensity of Eu^3+^, pointing to the formation of several UO_2_–Eu coordination species in solution (Figure 1). The addition of UO_2_^2+^ nitrate is first characterised by a strong increase of the overall Eu^3+^ emission intensity with, at maximum, a 6.4‐fold increase obtained at 613 nm in the presence of 0.5 equivalents of UO_2_^2+^. This observation clearly suggests the formation of a 2:1 EuL/uranyl(VI) species. After 0.5 equivalents, the Eu^3+^ emission intensity decreases, pointing to the formation of additional species in solution. Broad bands at around 530 nm, corresponding to UO_2_^2+^ emission, only became significant in the presence of an excess of 1 equivalent of UO_2_^2+^ (Figure S2). Linear regression analysis of the initial addition of UO_2_^2+^ to [EuL] provided limit of detection (LOD) values down to 12 μm (8.2×10^−5^ m [EuL], *λ*_exc_=320 nm). It however should be noted that here, neither the complex nor the titrations were desgined to maximise the LOD.

The spectral variations were analysed using the nonlinear regression analysis provided by SPECFIT (see the Supporting Information).^12^ The analysis confirmed the formation of two new species and the titrations were modelled, with the fitting procedure converging towards logarithmic values of 4.3±0.1 and 7.4±0.1 for *β_11_* and *β_21_*, respectively, corresponding to the formation of [(EuL)UO_2_] and [(EuL)_2_UO_2_] species. Excitation into the LMCT UO_2_^2+^ transition, at 420 nm (Figure S3), revealed a similar evolution.

During the titration, the intensity decays of Eu^3+^ (*λ*_em_=613 nm) were monitored with excitation at 280 nm and 340 nm (Table S1). In the absence of UO_2_^2+^, the excited state lifetime of [EuL] in TRIS buffer upon ligand excitation was 589 μs, in excellent agreement with previously reported data.^7^ For [U]/[Eu]<0.5, a bi‐exponential decay was obtained with lifetimes of *τ*_1_=340 μs and *τ*_2_=688 μs, in almost equal proportions. This behavior is likely due to the formation of an asymmetric 2:1 Eu^3+^/UO_2_^2+^species and points to the presence of two distinct coordination environments around Eu^3+^. From 0.6 equivalents and beyond, a short component corresponding to the 1:1 Eu^3+^/UO_2_^2+^species is observed with a lifetime of 180 μs.

Detailed examination of the contribution of each lifetime between 0 and 0.7 equivalents (*λ*_exc_=280 nm) shows that the 589 μs component reflects the disappearance of [EuL] according to the species distribution diagram in Figure S4 and the gradual increase of the 340 μs component (from 4 % to 54 %) corresponds well with the formation of the 2:1 species.

Significant changes were also observed by monitoring the UO_2_^2+^ lifetime (*λ*_exc_=303 nm, *λ*_em_=520 nm). The time‐resolved emission decay of UO_2_(NO_3_)_2_ was initially recorded in the same conditions and a mono‐exponential decay was observed with a luminescent lifetime of 1.9 μs, as expected for aqueous UO_2_^2+^ ions.^4^ A bi‐exponential decay was clearly observed for an EuL/U ratio of 1:0.25, showing a major component with τ_1_=379 ns (92 %) and a minor component *τ*_2_=54 ns (8 %). The obvious shortening of the lifetime of the UO_2_^2+^ fluorescence corroborates the depopulation of the UO_2_^2+^ excited states due to an intramolecular energy transfer. The relative populations of the two species are in strong agreement with the species distribution postulated. For the EuL/U ratio of 1:0.75, bi‐exponential decay was also observed, with a major component (*τ*_1_=379 ns, 79 %), accounting for the (EuL)_2_UO_2_ species, and a minor component (*τ*_2_=36 ns, 21 %), which can be attributed to the formation of the 1:1 complex. At a twofold excess of UO_2_^2+^ a bioexponential decay is observed, with the predominant species (*τ*_1_=1.9 μs, 98 %) being related to the presence of uncomplexed UO_2_^2+^. On the basis of these observations, the 379 ns lifetime was attributed to the [(EuL)_2_UO_2_] heterotrinuclear complex, while the heterodinuclear species, [(EuL)UO_2_], presented an average lifetime of 47 ns. Considering that the UO_2_^2+^‐based luminescence lifetime shortening is due to resonant energy transfer to Eu^3+^, it was possible to calculate U→Eu energy transfer efficiencies of 80 % in the [(EuL)_2_UO_2_] species and almost quantitative (97 %) in the [(EuL)UO_2_] dinuclear complex. The differences observed may be attributed to a stronger, essentially electrostatic interaction in the dinuclear species, as a result of the attraction of the positively charged UO_2_^2+^ cation with the negatively charged [EuL]^3/4−^ complex.

To gain insights into the polynuclear species formed in solution in the presence of UO_2_^2+^, we turned our attention to DFT calculations (Figure 2, Table S2). In this model, one oxygen atom of the UO_2_^2+^ group displays an electrostatic interaction to one of the Eu^3+^ centers (Eu–O=2.57 Å), while the second oxygen atom remains uncoordinated. Four oxygen atoms of phosphonate groups are coordinated to the UO_2_^2+^ ion with U–O distances of 2.21–2.22 Å. The two U=O distances are nearly identical (1.80, 1.81 Å), being close to those observed by EXAFS for UO_2_(CO_3_)_3_^4−^ in solution^13^ and other theoretical studies.^14^ The Eu–U distances are 4.12 Å for the Eu^3+^ complex coordinated through the O atom of the UO_2_^2+^ and 5.62 Å for the unbound uranyl oxygen atom.

**Figure 2:**
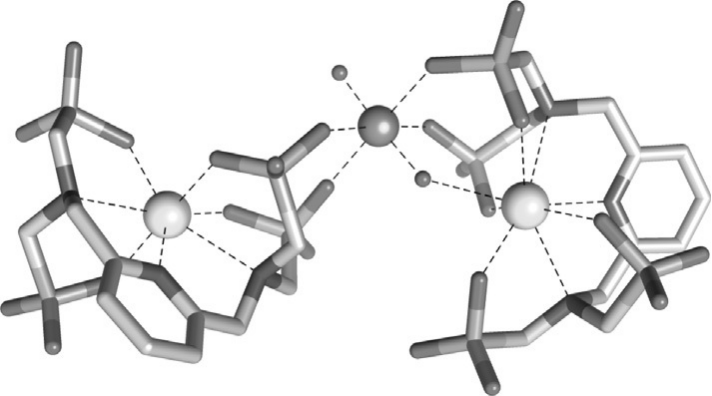
Optimised geometry of the [(EuL)_2_(UO_2_)]^8−^ system obtained with DFT calculations. (See the Supporting Information for computational details.)

DFT calculations were also performed on the dinuclear [(EuHL)(UO_2_)(H_2_O)_3_]^2−^ species (Figure S6, Table S3). The UO_2_^2+^ group is coordinated to the Eu^3+^ center with a Eu–O distance of 2.57 Å and a Eu–U distance of 4.02 Å. Two oxygen atoms of phosphonate groups coordinate to the UO_2_^2+^ ion (U–O=2.16, 2.20 Å), while three water molecules complete the equatorial coordination positions with relativley long U–O distances in the range 2.49–2.54 Å.^15^

Our DFT calculations should be taken with some care in view of the complexity of the systems under study, and the fact that our simplified model did not include explicit water molecules (bulk solvent effects were included using a polarizable continuum model). Nevertheless, they suggest that the polynuclear species formed upon UO_2_^2+^ addition are related to the coordination of phosphonate groups to the equatorial positions of UO_2_^2+^, likely resulting in two different Eu^3+^ environments. Such coordination is in excellent agreement with the luminescence lifetimes measured for the heterotrinuclear species. The two distinct lifetimes observed (340/690 μs) could correlate perfectly to two species with different hydration states as suggested by the calculations. One Eu^3+^ species is heptacoordinated by the ligand and fulfils its coordination by a water molecule, as is observed for the [EuL] complex itself,^7^ while coordination of the apical O atom of UO_2_^2+^ to the second Eu^3+^ centre likely prevents water coordination, resulting in an increased lifetime (*τ*=690 μs) compared to the [EuL] complex (*τ*=590 μs). Although Raman spectroscopy was attempted to characterise these interactions further, overlapping bands and weak signals precluded any definitive conclusion by this technique.

The solution assembly process was studied by ^1^H NMR spectroscopy in D_2_O (Figure S7). To avoid the complexity associated with paramagnetic contributions, the association behaviour of UO_2_(NO_3_)_2_ with the diamagnetic surrogate complex, [YL], was studied.^16^ The pattern and chemical shifts were similar to those observed for the previously studied lanthanum complex, pointing to a complex with *C*_2*v*_ symmetry and a coordination around Y^III^ in which the nitrogen atoms and two phosphonate functions form a quasi‐planar pentadentate chelating arrangement; the two remaining phosphonate moieties are coordinated on the upper and lower hemisphere of the complex, with the in‐plane and out‐of‐plane phosphonate functions in rapid exchange on the NMR timescale.^17^

Addition of UO_2_^2+^ results in a progressive decrease in intensity of the [YL] signals as a new set of peaks emerges. The new signals present significant downfield shifts with respect to the parent complex (Δ*δ*≈+0.5 to +0.7 ppm), except for the aromatic methylene bridges, which show a significant shift to higher fields (Δ*δ*≈−0.6 ppm). In contrast to the UV/Vis and emission spectroscopy titration, ^1^H NMR spectroscopy did not provide evidence for the formation of different heteronuclear species, suggesting they are in fast exchange under the conditions applied, even at lower temperatures (5 °C, data not shown). The relatively broad peaks of the new resonances compared with those of the [YL] complex are in line with this hypothesis. Additionally, the observation of up to five broad signals in the aliphatic region (Figure S7) suggests that the overall symmetry around the Y^III^ ion is decreased to *C_2_*, pointing to a rigidification of the structure upon UO_2_^2+^ interaction and slower in/out‐of‐plane exchange of the phosphonate functions.

Despite their widespread use as cation sensors, through both luminescence^8^, ^18^ and/or magnetic resonance responses,^19^ to the best of our knowledge there have been no reports of a molecular lanthanide long‐lived emissive complex that is responsive to UO_2_^2+^. This example adds to the scope of recent examples of energy transfer in molecular lanthanide(III) complexes,^20^ expanding applications into lanthanide–actinide interactions. Upon addition of UO_2_^2+^ to [EuL], our data indicate the formation of heteronuclear adducts in solution, accompanied by an appearance of characteristic UO_2_^2+^ transitions at 320 and 430 nm in the Eu^3+^ excitation spectra. Such transitions can only be due to resonant energy transfer from the UO_2_^2+^ ion to Eu^3+^, with energy transfer efficiencies up to 97 %. Multiplex sensing may also be feasible through resonance fluorescence measurements.^21^ While the unoptimised LOD presented here (ca. 12 μm) is higher than some previously reported (destructive) fluorescence sensors,^22^ it is significantly lower than commonly used X‐ray absorption techniques (ca. mm or ppm);^23^ further studies and ligand design should lead to lower detection limits for such phosphorescent sensors.

The complex used in this study was not designed to selectively bind UO_2_^2+^ and so, while other cations cannot cause the energy transfer presented, competing metal ions (e.g. Mg^2+^)^7^ may displace UO_2_^2+^ and lower the detection limit in actual environmental samples. However, up to 200 equivalents of environmentally ubiquitous Ca^2+^ ions have been shown not to significantly interact with [GdL].^7^ Higher specificity, in addition to the potential for time‐gated luminescence, should likely preclude interference from environmental chromophores, such as humic acid. Future incorporation of this strategy with a small‐molecule Eu^3+^ complex specifically designed with a high UO_2_^2+^ binding constant would result in a powerful and relatively inexpensive tool that could be developed to selectively detect environmental UO_2_^2+^ in situ in contaminated groundwater sites.

## Conflict of interest

The authors declare no conflict of interest.

## Supporting information

As a service to our authors and readers, this journal provides supporting information supplied by the authors. Such materials are peer reviewed and may be re‐organized for online delivery, but are not copy‐edited or typeset. Technical support issues arising from supporting information (other than missing files) should be addressed to the authors.

Supplementary
