# Supplementary material for: Sensing Uranyl(VI) Ions by Coordination and Energy Transfer to a Luminescent Europium(III) Complex
Source: Angew Chem Int Ed Engl. 2018 Jun 29;57(31):9921–4. doi: 10.1002/anie.201805316 (PMC6099227; doi:10.1002/anie.201805316)
Supplement: Supplementary file 1 — Supplementary [file ANIE-57-9921-s001.pdf]

Angewandte Chemie
Eine Zeitschrift der Gesellschaft Deutscher Chemiker

Supporting Information

Sensing Uranyl(VI) Ions by Coordination and Energy Transfer to a Luminescent Europium(III) Complex

Peter Harvey, Aline Nonat, Carlos Platas-Iglesias, Louise S. Natrajan, and Loïc J. Charbonnière**

anie_201805316_sm_miscellaneous_information.pdf

Supporting Information

Figure S1. UV-Vis absorption spectra of the titration of [EuL] upon addition of uranyl nitrate ([EuL] = 8.2×10^{-5} M, 0.01 M TRIS buffer, pH 7.4).

Figure S2. Evolution of the excitation spectra (λ_{em} = 613 nm) during the titration of a solution of [EuL] upon addition of uranyl nitrate ([EuL] = 8.2×10^{-4} M, 0.01 M TRIS buffer, pH 7.4), indicating growth of a distinct uranyl(VI) excitation bands at 320/430 nm.

Figure S3. Emission spectra (λ_{ex} = 420 nm) for the addition of uranyl nitrate to [EuL] (8.2×10^{-4} M [Eu], 0.01 M TRIS buffer, pH 7.4).

Figure S4. Speciation diagram of the species formed during the titration of [EuL] by $UO_2(NO_3)_3$ ([EuL] = 8.2×10^{-4} M, 0.01 M TRIS buffer, pH 7.4, [EuL], red; $[(EuL)_2UO_2]$, green, and $[(EuL)UO_2]$, blue).

Figure S5. Calculated spectra of the species formed during the titration of [EuL] by $UO_2(NO_3)_3$ ([EuL] = 8.2×10^{-4} M, 0.01 M TRIS buffer, pH 7.4, [EuL], red; $[(EuL)_2UO_2]$, green, and $[(EuL)UO_2]$, blue).

Figure S6. Optimized geometry of the $[(EuHL)(UO_2)(H_2O)_3]^{2-}$ system obtained with DFT calculations.

Figure S7. ^{1}H-NMR spectra of a solution of [YL] upon addition of $UO_2(NO_3)_2$ in D_2O.

Table S1. Eu excited state lifetimes (λ_{em}= 613 nm) and the corresponding populations upon excitation at 280 nm and 340 nm, upon addition of uranyl nitrate in a EuL solution (pH 7.4 (TRIS/HCl 0.01M, C_{EuL} = 8.2×10^{-4}M)

Table S2. Optimised Cartesian coordinates (Å) of $[(EuL)_2(UO_2)]^{8-}$, TPSSh, 0 imaginary frequencies.

Table S3. Optimised Cartesian coordinates (Å) of $[(EuL)(UO_2)(H_2O)_3]^{2-}$, TPSSh, 0 imaginary frequencies.

Table S4. Relative intensities of the emission bands observed upon different excitation frequencies. Values reflect areas under designated emission bands corresponding to either uranyl(VI) or europium(III).

Figure S8. Plot of the Eu(III) $^5D_0 \rightarrow {}^7F_2$ to $^5D_0 \rightarrow {}^7F_2$ integrated emission ratio ($\Delta J=2/\Delta J=1$) integrated emission against the number of equivalents of uranyl(VI) added. Typically, the ratio of these bands indicates the site symmetry around the Eu(III) ion, with a ratio >8 indicating that Eu(III) lies in a low symmetry environment and a ratio of <0.7 indicating the Eu(III) ion lies in a centrosymmetric coordination environment.[12]

Experimental details

The Eu and Y complexes of L were synthesized as previously reported.[1]

All NMR spectra were recorded on a Bruker Avance 400 spectrometer (Manchester). The NMR spectrometer was controlled remotely using Bruker Topspin 2.1 or 3.1 software. Chemical shifts are reported in parts per million relative to TMS.

UV-Vis absorption spectra were recorded on a Specord 205 (Analytik Jena) or a Perkin–Elmer lambda 950 spectrometer. Steady state emission and excitation spectra were recorded on an Edinburgh Instrument FP920 Phosphorescence Lifetime Spectrometer equipped with a 5 watt microsecond pulsed xenon flashlamp (with single 300 mm focal length excitation and emission monochromators in Czerny Turner configuration) and a red sensitive photomultiplier in peltier (air cooled) housing, (Hamamatsu R928P) and a liquid nitrogen cooled nIR photomultiplier (Hamamatsu). Lifetime data were recorded following excitation with either the microsecond flashlamp, an EPL 375 and EPL 405 picosecond pulsed diode laser (Edinburgh Instruments), or a picosecond pulsed Supercontinuum laser at 420 nm (Fianium) using time correlated single photon counting (PCS900 plug-in PC card for fast photon counting). Lifetimes were obtained by tail fit on the data

obtained or by a reconvolution fit using a solution of Ludox® in the scatterer, and quality of fit judged by minimization of reduced chi-squared and residuals squared.

For a typical spectrophotometric titration experiment, to 2 mL of a 10^{-3}-10^{-4} M solution of [EuL] (0.01 M TRIS/HCl, pH 7.4) in a 1 cm quartz cell were added increasing amounts of a 10 times more concentrated solution of $(UO_2)(NO_3)_2$ in the same buffer. After addition of each aliquots, the UV-Vis absorption spectra and emission spectra were recorded. At different point of the titration, kinetic experiments were performed by repeating the measurements after few minutes, to ensure that the thermodynamic equilibrium was reached. The spectrophotometric data were analyzed with the Specfit program,[2] which adjusts the absorptivities and the stability constants of the species formed at equilibrium. Specfit uses factor analysis to reduce the absorbance matrix and to extract the eigenvalues prior to the multi-wavelength fit of the reduced data set according to the Marquardt algorithm.[3] The titrations were modeled with the following equilibria (charges are omitted for simplicity):

EuL + UO_2 □ [(EuL)UO_2] $\qquad$ $\beta_{11} = \frac{[(EuL)UO_2]}{[EuL]\times[UO_2]}$ $\qquad$ (1)

2 EuL + UO_2 □ [$(EuL)_2UO_2$] $\qquad$ $\beta_{21} = \frac{[(EuL)_2UO_2]}{[EuL]^2\times[UO_2]}$ $\qquad$ (2)

The U □ Eu energy transfer efficiency, $□_{U□Eu}$, was calculated using the relationship :

$□_{U□Eu}$ = 1 - τ_{UEu}/τ_U $\qquad$ (3)

where τ_{UEu} stands for the lifetime of uranyl in the presence of the acceptor, the europium cations in the present complexes.

Limit of detection (LOD) was calculated from linear regression analysis of the Eu(III) emission intensity changes upon addition of uranyl(VI) at various wavelengths *via* the following equation;

$$LOD = 3s_{xy}/m$$

where s_{xy} is the standard error and m is the gradient for the plot $y = mx + c$ (where y is the Eu(III) emission intensity; x is uranyl concentration). Regression analysis with the LINEST function in Microsoft Excel was performed on linear regions of emission intensity changes and/or Stern-Volmer plots to determine the lowest LOD with this system. Lowest LOD (12 μM) was calculated from the increase in [EuL] emission upon 320 nm excitation (8.2 x 10^{-4}M [EuL], 0-0.6 equivalents of UO_2^{2+}).

Computational details.

All calculations were performed employing DFT within the hybrid meta-GGA approximation with the TPSSh exchange-correlation functional,[4] and the Gaussian 09 package (Revision D.01).[5] Geometry optimizations were performed in aqueous solution by using the large-core relativistic effective core potential (LCRECP) of Dolg *et al.* and the related (7s6p5d)/[5s4p3d]-GTO valence basis set for Eu,[6] which includes 46+$4f^6$ electrons in the core for Eu(III) leaving the outermost 11 electrons (5s, 5p, 5d and 6s) to be treated explicitly. Similarly, U was treated with a 5f-in-core RECP and a (7s6p5d2f1g)/[6s5p4d2f1g] valence basis set.[7] All other atoms were described using the standard 6-31G(d) basis set. Since LCRECPs include the 4f electrons in the core for Eu, calculations were conducted on a pseudo-singlet state configuration. No symmetry constraints have been imposed during the optimizations. The default values for the integration grid (75 radial shells and 302 angular points) and the SCF energy convergence criteria (10^{-8}) were used in all calculations. The stationary points found on the potential energy surfaces as a result of the geometry optimizations have been tested to represent energy minima rather than saddle points via frequency analysis. Bulk solvent effects (water) were evaluated by using the polarizable continuum model (PCM). In particular, we used the integral equation formalism (IEFPCM)[8] variant as implemented in Gaussian 09. The universal force field radii (UFF)[9] scaled by a factor of 1.1 were used to define the solute cavities. Molecular graphics were generated using USCF Chimera (version 1.8).

It is well known that lanthanide(III) complexes containing phosphonate groups can be protonated at relatively high pH values,[10] with protonation constants of logK_1 = 9.4 and logK_1 = 7.5 having been determined for [EuL].[1] Thus, theoretical DFT calculations were also performed on the $[(EuHL)_2(UO_2)]^{6-}$ system. The optimized geometry of the protonated complex provides a U(VI) coordination environment virtually identical to that obtained for the $[(EuL)_2(UO_2)]^{8-}$ complex, the main difference between the two structures concerning the slight lengthening of the Eu-O distances involving the protonated phosphonates (~0.1 Å). This result is in line with previous studies, which showed that phosphonate groups usually remain coordinated to lanthanide ions when protonated.[11]

Experimental details

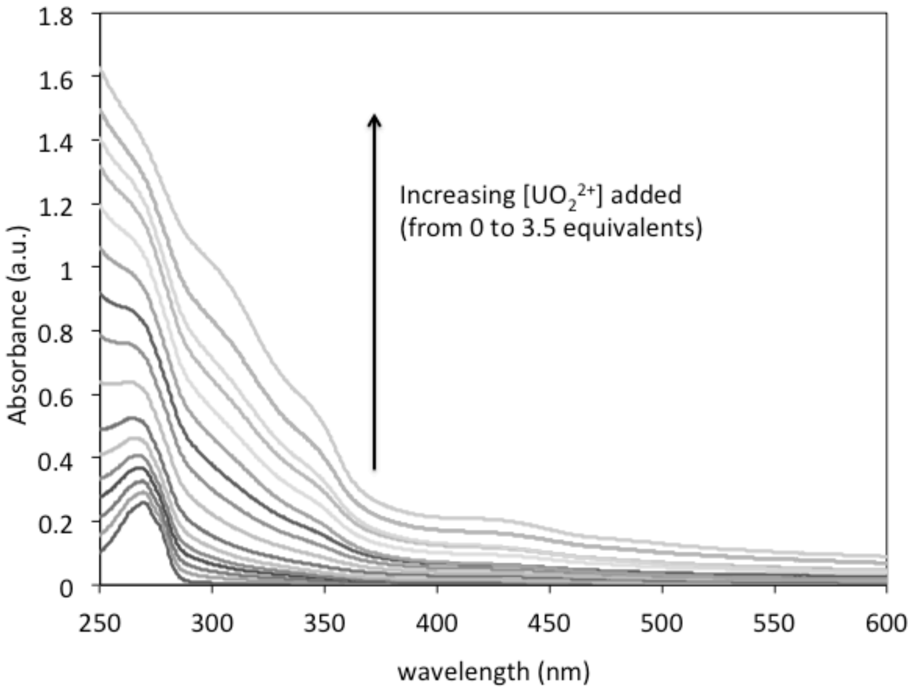

Figure S1. UV-vis absorption spectra of the titration of [EuL] upon addition of uranyl nitrate ([EuL] = 8.2 x 10^{-5} M, 0.01 M TRIS buffer, pH 7.4).

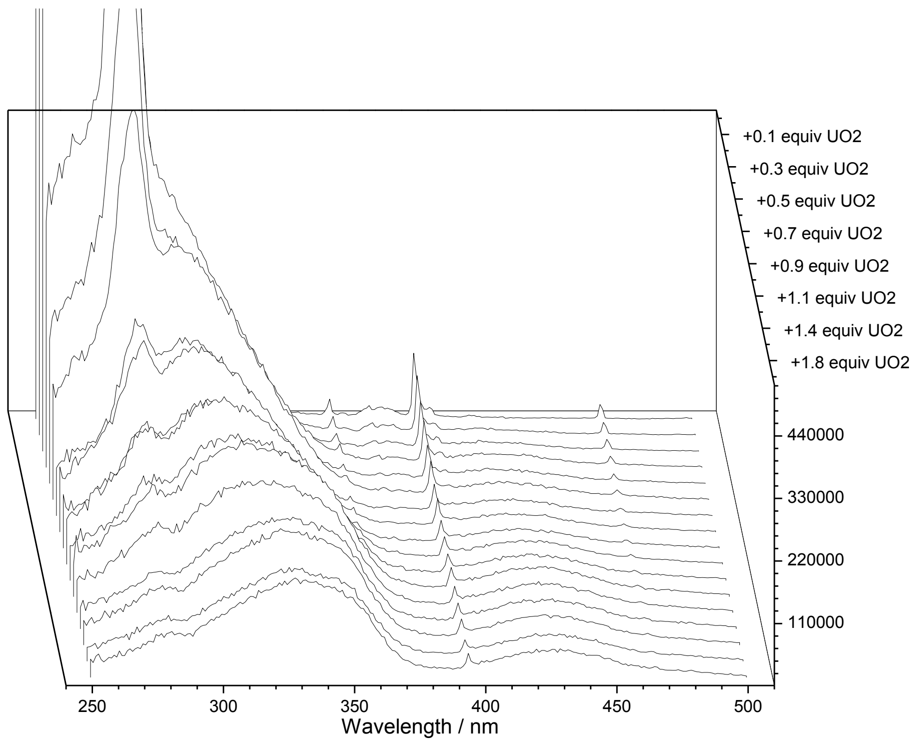

Figure S2. Evolution of the excitation spectra (λ_{em} = 613 nm) during the titration of a solution of [EuL] upon addition of uranyl nitrate ([EuL] = 8.2 x 10^{-4} M, 0.01 M TRIS buffer, pH 7.4), indicating growth of a distinct uranyl(VI) excitation bands at 320/430 nm.

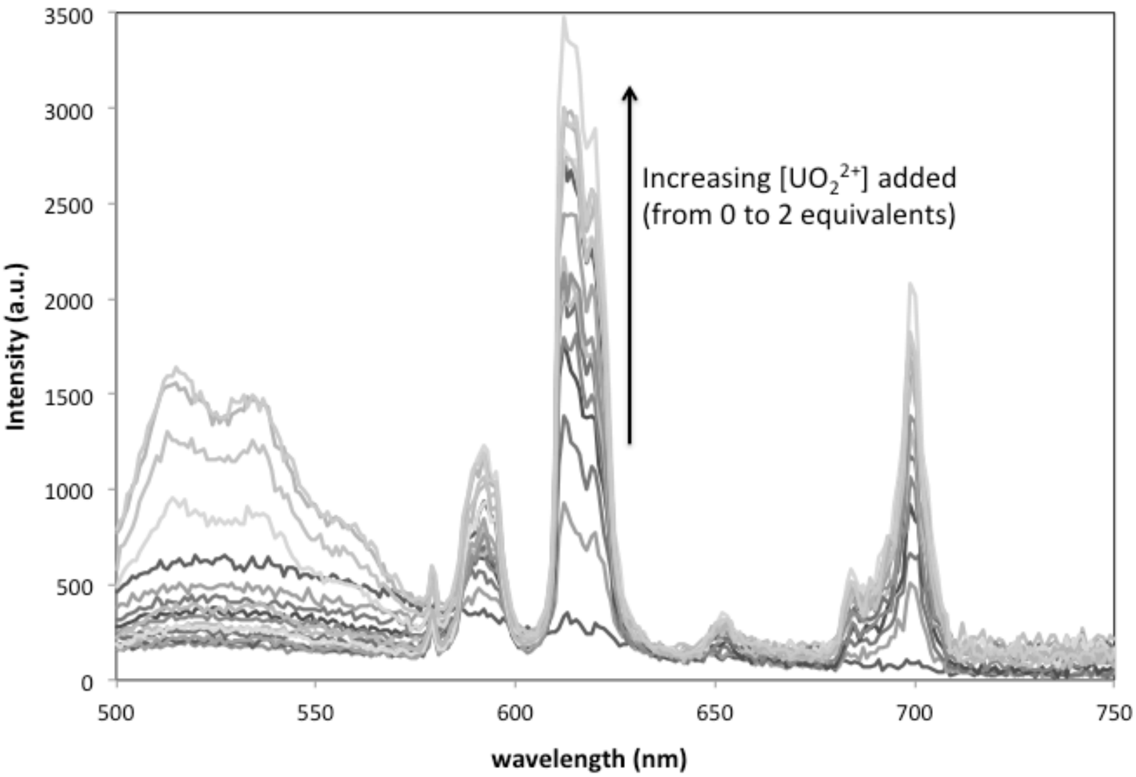

Figure S3. Emission spectra (λ_{exc} = 420 nm) for the addition of uranyl(VI) nitrate to [EuL] complex (8.2 x 10^{-4} M [Eu], 0.01 M TRIS buffer, pH 7.4). Note minimal Eu(III) emission at this excitation wavelength before addition of uranyl(VI).

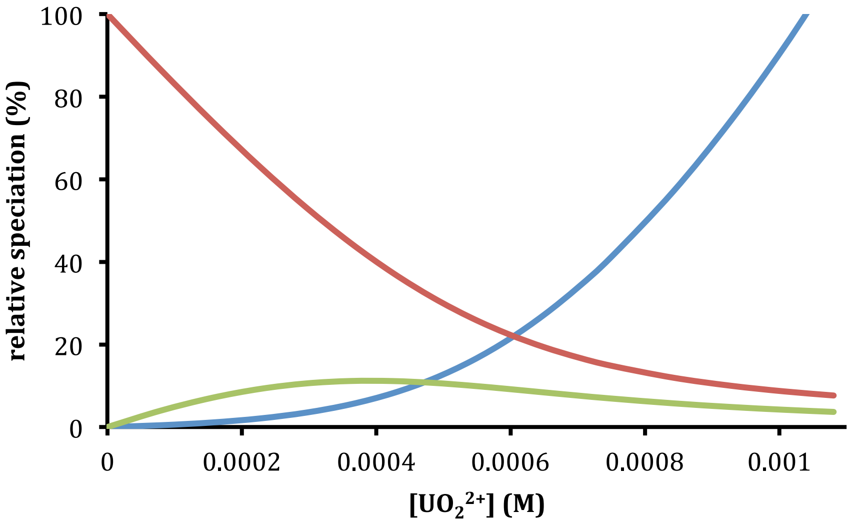

Figure S4. Speciation diagram of the species formed during the titration of [EuL] by $UO_2(NO_3)_3$ ([EuL] = 8.2 x 10^{-4} M, 0.01 M TRIS buffer, pH 7.4, [EuL], red; $[(EuL)_2UO_2]$, green, and $[(EuL)UO_2]$, blue).

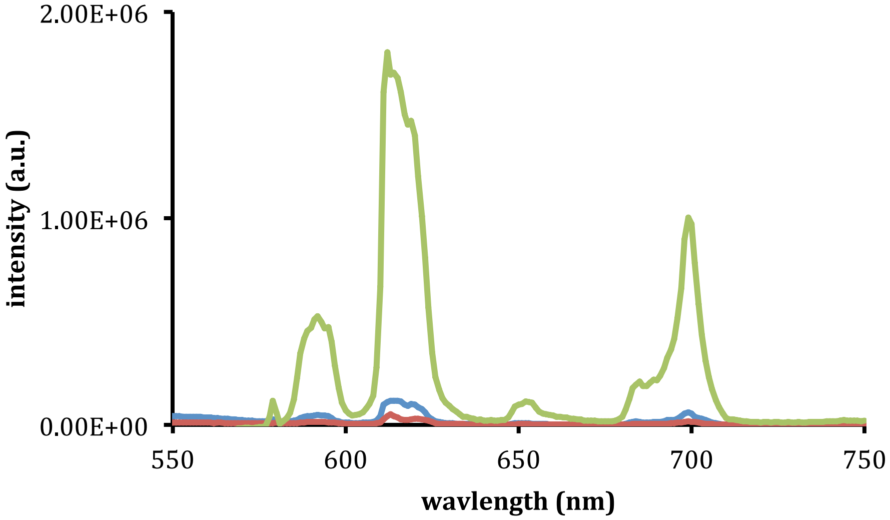

Figure S5. Calculated spectra of the species formed during the titration of [EuL] by $UO_2(NO_3)_3$ ([EuL] = 8.2 x 10^{-4} M, 0.01 M TRIS buffer, pH 7.4, [EuL], red; $[(EuL)_2UO_2]$, green, and $[(EuL)UO_2]$, blue).

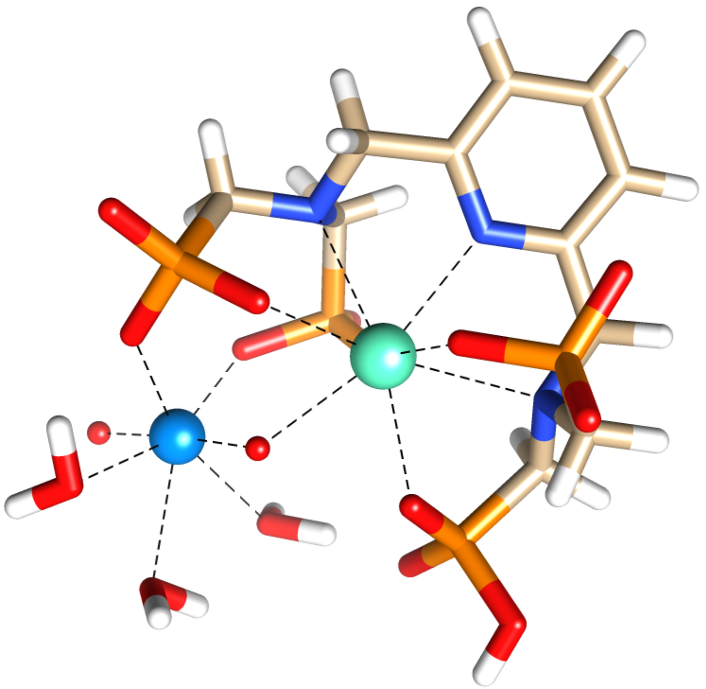

Figure S6. Optimized geometry of the $[(EuHL)(UO_2)(H_2O)_3]^{2-}$ system obtained with DFT calculations.

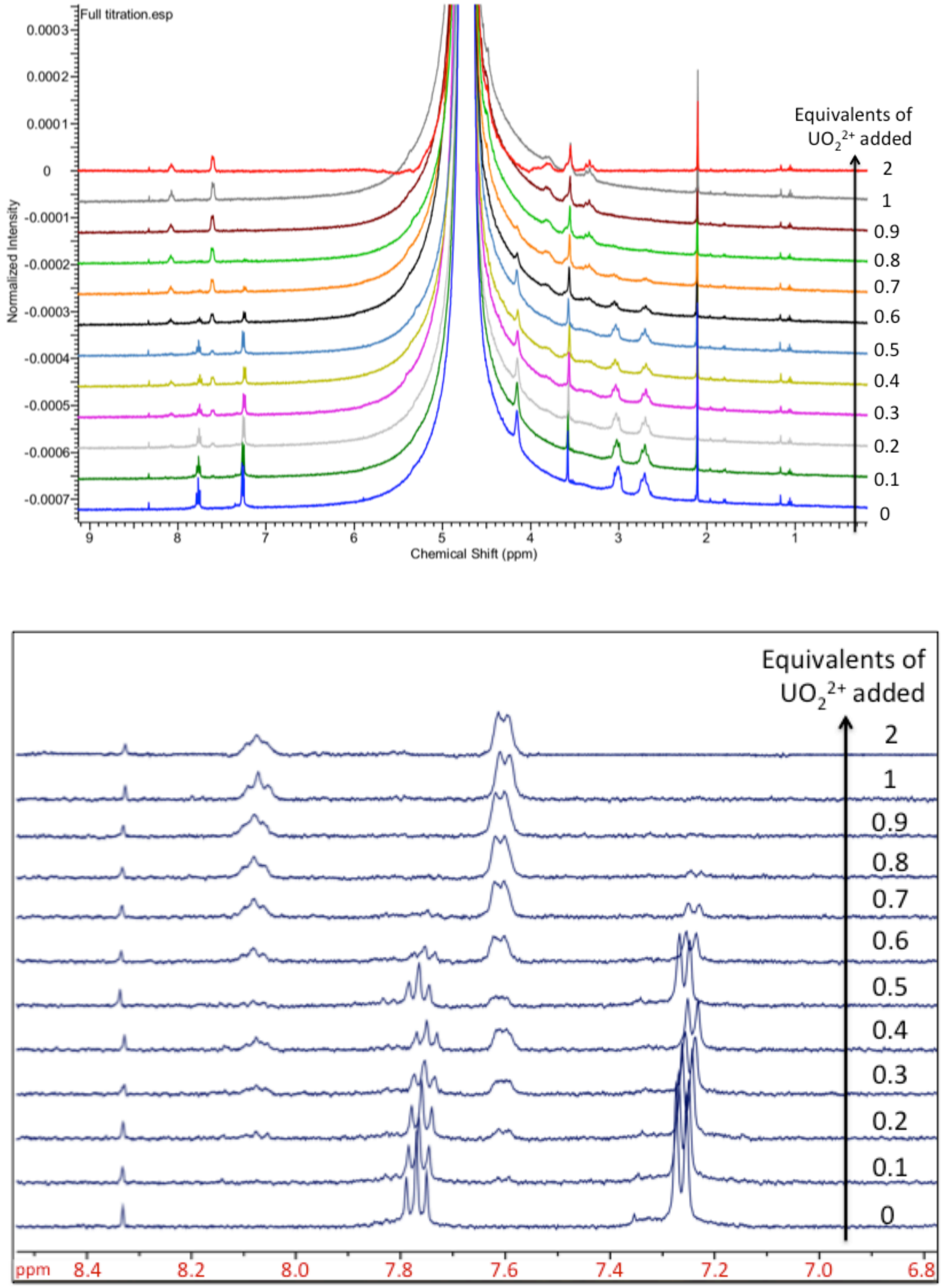

Figure S7. ^{1}H-NMR spectra of a solution of [YL] (0.82 mM) upon addition of $UO_2(NO_3)_2$ in D_2O (298 K, 400 MHz, 0.01 M d-TRIS). Lower figure shows expansion of the aromatic region.

Table S1. Eu(III) excited state lifetimes (λ_{em}= 613 nm) upon excitation at 280 nm and 340 nm, upon addition of uranyl nitrate in a EuL solution (pH 7.4 (TRIS/HCl 0.01M, C_{EuL} = 8.2×10^{-4}M)

	λ_{exc} = 280 nm		λ_{exc} = 340 nm	
**n = [UO_2]/[EuL]**	τ_i (i=1-3) (μs)[§]	α_i (%)	τ_i (i=1-3) (μs)[§]	α_i (%)
**0**	589	100	524 ± 3	100
**0.1**	*589*	65	343 ± 16	41
	340	4	688 ± 27	59
	688	31		
**0.2**	*589*	49	334 ± 9	45
	340	10	688 ± 19	55
	688	41		
**0.3**	*589*	42	328 ± 8	45
	340	16	666 ± 16	55
	688	42		
**0.4**	*589*	50	321 ± 6	47
	340	33	688 ± 13	53
	688	17		
**0.5**	*589*	20	315 ± 5	50
	340	32	669 ± 13	50
	688	48		
**0.6**	*589*	10	*335*	51
	340	39	*688*	45

	688	51	176 ± 9	4
**0.7**	*340*	54	*335*	57
	688	46	*688*	63
			175	36
**0.9**	*340*	53	*335*	53
	688	41	*688*	34
	163 ± 9	6	*175*	13
**1.0**	*340*	4	*335*	51
	688	51	*688*	36
	155 ± 12	45	*175*	13
**1.2**	*340*	50	*335*	49
	688	42	*688*	35
	167 ± 9	8	*175*	16
**1.6**	*340*	48	*335*	48
	688	44	*688*	39
	166	8	*175*	13
**2.0**	*340*	45	*335*	44
	*688**	47	*688*	44
	166	8	*175*	12

[§]*Values in italic have been fixed for the fitting.*

*Note that convoluted variations are observed in the 688 μs component, suggesting the formation of an additional species with a similar lifetime in this range.

Table S2. Optimised Cartesian coordinates (Å) of $[(EuL)_2(UO_2)]^{8-}$, TPSSh, 0 imaginary frequencies.

C	7.03985300	-1.14649400	0.34575900
C	8.36284900	-1.36466500	0.73785400
H	8.88234200	-2.26389400	0.42144500
C	6.30030000	-2.14806700	-0.50939300
N	4.89098400	-2.29637700	-0.09765700
C	4.22825800	-3.24836000	-1.02309200
H	3.49875100	-3.82544900	-0.45146300
H	4.96329100	-3.95800400	-1.43380300
C	4.75131800	-2.72997400	1.31562200
H	4.76971700	-3.82654100	1.40789800
H	5.59939000	-2.34174000	1.88824100
H	6.29158800	-1.80950200	-1.55402000
H	6.83185700	-3.11469700	-0.48420900
P	3.30266700	-2.41311200	-2.40463000
O	3.67697100	-0.90324800	-2.30952300
O	3.58737400	-3.08770000	-3.73225100
O	1.78436600	-2.64050600	-1.94469700
P	3.24487800	-1.98292300	2.08262200
O	2.03389100	-2.57271900	1.21586400
O	3.40421700	-0.45616500	1.80787900
O	3.15956100	-2.39940000	3.53796900
H	4.26988300	3.96753300	2.39773300
C	3.93242700	3.07108100	1.84586400
N	5.06365900	2.45146100	1.10031200
H	3.60232800	2.33245800	2.58533100
P	2.47591600	3.47659600	0.73760800
C	6.06922400	1.96124100	2.05300200
C	5.64715500	3.43230900	0.13950300
O	2.60105800	2.31258300	-0.33650000
O	1.22782700	3.32968100	1.62084400
O	2.69875000	4.85751600	0.10650900
C	6.94131400	0.84853700	1.52040700
H	5.52918100	1.55050200	2.91546900
H	6.69977600	2.78361000	2.43126000
H	4.81692700	4.07141400	-0.17412700
H	6.36796000	4.08154100	0.67025600
P	6.40336700	2.73484100	-1.41704600
C	8.26313400	0.70012900	1.95791400
N	6.34436500	-0.05791500	0.72736000
O	6.28237700	3.86789500	-2.44588400
O	5.40790000	1.53281700	-1.74921900
O	7.82666100	2.24053000	-1.12848800
C	8.98649600	-0.41867800	1.55454700
H	8.70621700	1.45198400	2.60391300

H	10.01330700	-0.56121600	1.87892900
U	0.53055500	-1.89699100	-0.27456600
O	1.48128900	-0.35885600	-0.40177700
O	-0.31408400	-3.48231200	-0.17418000
C	-4.13281500	3.08565200	-0.72666300
C	-4.22727800	4.25860800	-1.47746200
H	-3.53536800	5.07573000	-1.29972500
C	-3.14045000	2.95008600	0.40529400
N	-2.55615800	1.59264200	0.48126600
C	-1.80821500	1.45414600	1.76427700
H	-0.82820000	1.95399300	1.71691800
H	-2.39782000	1.96042400	2.53842100
C	-1.67354300	1.34936500	-0.70303300
H	-0.65713500	1.15029500	-0.35305500
H	-1.63189300	2.26255700	-1.30846300
H	-3.67505900	3.13239800	1.34626200
H	-2.35868000	3.72094600	0.31707500
P	-1.68853500	-0.29357800	2.34214500
O	-3.14931900	-0.82824600	2.15734200
O	-1.15061200	-0.31060900	3.75788800
O	-0.73142100	-1.03391800	1.31712200
P	-2.15483700	-0.03914700	-1.86115900
O	-0.99045700	-1.11166700	-1.68757000
O	-3.47262600	-0.66797600	-1.31055800
O	-2.22403500	0.49850700	-3.27766300
H	-8.20458000	-1.43983300	-1.90011300
C	-7.21068900	-1.38227100	-1.42158900
N	-6.93759000	0.00729400	-0.94905800
H	-6.46481600	-1.61207700	-2.19126100
P	-7.02205600	-2.68548700	-0.09318600
C	-6.75866000	0.89506600	-2.11251000
C	-8.04831600	0.45790500	-0.05207100
O	-5.67481400	-2.18651800	0.60912800
O	-6.82886300	-4.00163900	-0.84778800
O	-8.21224100	-2.60880800	0.87288300
C	-5.92329400	2.12918900	-1.85330600
H	-6.23610800	0.31836500	-2.88635600
H	-7.73076800	1.18945900	-2.54122300
H	-8.39357000	-0.45208100	0.45005700
H	-8.88564200	0.83502600	-0.66650400
P	-7.63352400	1.66506500	1.31140300
C	-6.08854100	3.27754100	-2.63811100
N	-4.95588500	2.03647900	-0.92545900
O	-8.72624900	1.44100800	2.36319100
O	-6.21911300	1.09757800	1.81368900
O	-7.51431000	3.08478900	0.74940000
C	-5.22501400	4.35379500	-2.45021600

H	-6.88031500	3.31762600	-3.37989300
H	-5.32553900	5.25370600	-3.04991300
Eu	3.92517100	0.44385200	-0.35409200
Eu	-4.73053100	-0.11636100	0.57372400

E(RTPSSh) = -5995.9493883 Hartree
Zero-point correction = 0.617301
Thermal correction to Energy = 0.682787
Thermal correction to Enthalpy = 0.683731
Thermal correction to Gibbs Free Energy = 0.518135
Sum of electronic and zero-point Energies = -5995.332087
Sum of electronic and thermal Energies = -5995.266601
Sum of electronic and thermal Enthalpies = -5995.265657
Sum of electronic and thermal Free Energies = -5995.431254

Table S3. Optimised Cartesian coordinates (Å) of $[(EuHL)(UO_2)(H_2O)_3]^{2-}$, TPSSh, 0 imaginary frequencies.

C	2.88396700	-2.65931800	0.61258200
C	3.98806900	-3.42414800	0.98707700
H	3.94449700	-4.50712400	0.93005100
C	1.61319200	-3.31835200	0.12838700
N	0.39918900	-2.57102600	0.52204800
C	-0.77753300	-3.24909200	-0.08450500
H	-1.61539000	-3.17659800	0.61104700
H	-0.56655200	-4.31694700	-0.23999100
C	0.23952400	-2.45608200	1.99492300
H	-0.28773000	-3.32305200	2.41717300
H	1.23109900	-2.41589500	2.45644800
H	1.61144900	-3.36301800	-0.96878900
H	1.57900300	-4.35678500	0.49523000
P	-1.31159300	-2.46383900	-1.67673200
O	-0.18993300	-1.43940300	-2.02489600
O	-1.65328700	-3.48831500	-2.73220900
O	-2.64399200	-1.67595100	-1.19134900
P	-0.56517800	-0.85017400	2.42194200
O	-1.97701600	-0.88832800	1.63658000
O	0.33216500	0.21037400	1.72250100
O	-0.75584500	-0.72720100	3.91486600
H	2.92805400	3.42327200	1.71033800
C	2.24893300	2.60369000	1.42966600
N	2.92943700	1.56258200	0.61672200
H	1.87101300	2.13054700	2.33870400
P	0.77390100	3.27057100	0.53808700
C	3.85656900	0.79446800	1.47304000
C	3.64907000	2.17966200	-0.53504300
O	0.61583700	2.40228900	-0.71671100
O	-0.44315400	3.46953600	1.44100500
O	1.30464100	4.76639700	0.09409000
C	3.96866900	-0.68099800	1.16232500
H	3.48319900	0.85205800	2.50280100
H	4.85804900	1.24711700	1.47480900
H	2.98471500	2.93583200	-0.95763000
H	4.55642900	2.69578100	-0.17649800
P	4.04729800	0.98987600	-1.91031500
C	5.11969000	-1.38204800	1.54623500
N	2.87954700	-1.30978300	0.68492400
O	4.33342100	1.85972900	-3.13490900
O	2.64585200	0.22142200	-2.04924500
O	5.16026100	0.05263000	-1.43708400
C	5.13424200	-2.76824700	1.44529700

H	5.98143600	-0.83832200	1.91999400
H	6.01371300	-3.33499200	1.73630100
U	-3.01365300	0.11415000	0.02449000
O	-1.49725600	0.72531500	-0.68106100
O	-4.58527800	-0.38999500	0.67072600
O	-2.60528800	2.04272700	1.54528500
O	-4.06158100	0.25398300	-2.28195700
Eu	0.96734400	0.01947400	-0.54580800
H	-3.79263900	-0.65445100	-2.54045500
H	-5.03521800	0.27557400	-2.32459900
H	-1.69256900	2.52264100	1.48005700
H	-2.81057200	1.95914000	2.49334700
H	0.55313000	5.38849400	0.09576400
O	-4.11473500	2.31980100	-0.57560600
H	-3.79611700	2.72774700	-1.40185300
H	-3.77416300	2.87417900	0.15993200

-

E(RTPSSh) = -3328.4938891 Hartree
Zero-point correction = 0.396497
Thermal correction to Energy = 0.438996
Thermal correction to Enthalpy = 0.439941
Thermal correction to Gibbs Free Energy = 0.324446
Sum of electronic and zero-point Energies = -3328.097393
Sum of electronic and thermal Energies = -3328.054893
Sum of electronic and thermal Enthalpies = -3328.053948
Sum of electronic and thermal Free Energies = -3328.169443

Table S4. Relative intensities of the emission bands observed upon different excitation frequencies. Values reflect areas under designated emission bands corresponding to either uranyl(VI) or europium(III).

	$[UO_2]$ equivalents	UO_2 emission	Δ*J* = 0	Δ*J* = 1	Δ*J* = 2	Δ*J* = 3	Δ*J* = 4
λ_{ex} = 280 nm	0	6.14E+04	5.42E+04	6.40E+05	3.25E+06	1.18E+05	1.24E+06
	0.1	3.93E+04	3.60E+04	4.58E+05	2.22E+06	8.45E+04	8.90E+05
	0.2	2.72E+04	2.46E+04	3.44E+05	1.58E+06	6.06E+04	6.63E+05
	0.3	1.89E+04	1.44E+04	2.12E+05	9.64E+05	3.68E+04	4.05E+05
	0.4	1.73E+04	1.10E+04	1.67E+05	7.39E+05	2.81E+04	3.17E+05
	0.5	1.46E+04	7.02E+03	1.09E+05	4.76E+05	1.82E+04	2.05E+05
	0.6	1.21E+04	4.51E+03	7.21E+04	3.02E+05	1.16E+04	1.29E+05
	0.7	1.31E+04	3.69E+03	6.15E+04	2.54E+05	1.01E+04	1.11E+05
	0.8	1.30E+04	3.06E+03	4.93E+04	1.97E+05	7.83E+03	8.61E+04
	0.9	3.02E+04	2.54E+03	3.68E+04	1.40E+05	6.51E+03	6.80E+04
	1	1.68E+04	2.28E+03	3.39E+04	1.37E+05	5.67E+03	5.98E+04
	1.1	2.17E+04	2.32E+03	3.31E+04	1.30E+05	5.51E+03	5.58E+04
	1.2	3.27E+04	2.43E+03	3.35E+04	1.32E+05	5.97E+03	6.18E+04
	1.4	4.07E+04	1.72E+03	2.04E+04	7.77E+04	3.51E+03	3.22E+04
	1.6	5.23E+04	1.87E+03	2.03E+04	7.47E+04	3.40E+03	3.33E+04
	1.8	5.37E+04	1.83E+03	1.86E+04	6.63E+04	2.78E+03	2.93E+04
	2	6.22E+04	1.89E+03	1.78E+04	6.58E+04	3.15E+03	3.18E+04
λ_{ex} = 320 nm	0	9.71E+04	2.83E+03	1.37E+04	3.93E+04	3.02E+03	1.60E+04
	0.1	6.66E+04	2.82E+03	2.93E+04	1.02E+05	5.25E+03	5.00E+04
	0.2	5.31E+04	3.38E+03	4.43E+04	1.61E+05	7.66E+03	7.93E+04
	0.3	4.49E+04	3.45E+03	4.86E+04	1.81E+05	8.36E+03	8.66E+04
	0.4	4.15E+04	3.48E+03	5.15E+04	1.95E+05	9.11E+03	9.22E+04
	0.5	3.65E+04	3.45E+03	5.11E+04	1.97E+05	9.04E+03	9.20E+04
	0.6	3.19E+04	3.08E+03	4.75E+04	1.82E+05	8.44E+03	8.56E+04
	0.7	2.80E+04	2.84E+03	4.46E+04	1.71E+05	7.99E+03	7.93E+04
	0.8	2.88E+04	2.87E+03	4.32E+04	1.62E+05	7.75E+03	7.70E+04
	0.9	3.25E+04	2.75E+03	3.93E+04	1.48E+05	6.55E+03	7.03E+04
	1	4.12E+04	2.85E+03	3.94E+04	1.46E+05	6.51E+03	6.89E+04
	1.1	5.46E+04	3.17E+03	4.20E+04	1.53E+05	6.85E+03	7.16E+04
	1.2	8.82E+04	3.66E+03	4.16E+04	1.42E+05	6.18E+03	6.24E+04
	1.4	1.34E+05	4.17E+03	4.03E+04	1.40E+05	6.24E+03	6.60E+04
	1.6	1.70E+05	4.48E+03	3.77E+04	1.26E+05	5.66E+03	5.69E+04
	1.8	2.14E+05	5.24E+03	4.13E+04	1.36E+05	6.07E+03	6.37E+04
	2	2.34E+05	5.24E+03	3.73E+04	1.17E+05	5.38E+03	5.29E+04
λ_{ex} = 340 nm	0	1.04E+05	2.50E+03	8.72E+03	1.46E+04	1.96E+03	6.16E+03
	0.1	7.57E+04	2.24E+03	1.47E+04	4.23E+04	3.01E+03	2.06E+04

	0.2	6.23E+04	2.23E+03	1.98E+04	6.60E+04	3.73E+03	3.23E+04
	0.3	5.40E+04	2.22E+03	2.34E+04	8.20E+04	4.48E+03	4.04E+04
	0.4	4.83E+04	2.31E+03	2.53E+04	9.21E+04	4.94E+03	4.48E+04
	0.5	4.39E+04	2.35E+03	2.79E+04	1.04E+05	5.52E+03	5.00E+04
	0.6	3.78E+04	2.26E+03	2.65E+04	9.88E+04	5.66E+03	4.94E+04
	0.7	3.30E+04	2.07E+03	2.63E+04	9.68E+04	5.48E+03	4.85E+04
	0.8	3.02E+04	2.10E+03	2.80E+04	1.05E+05	5.57E+03	5.18E+04
	0.9	3.36E+04	2.32E+03	3.01E+04	1.13E+05	5.71E+03	5.62E+04
	1	4.29E+04	2.69E+03	3.37E+04	1.24E+05	6.02E+03	5.94E+04
	1.1	5.29E+04	2.92E+03	3.50E+04	1.29E+05	6.04E+03	6.20E+04
	1.2	7.67E+04	3.30E+03	3.81E+04	1.37E+05	6.23E+03	6.48E+04
	1.4	1.30E+05	3.88E+03	3.67E+04	1.25E+05	5.76E+03	5.76E+04
	1.6	2.04E+05	4.97E+03	3.93E+04	1.28E+05	5.92E+03	6.04E+04
	1.8	2.10E+05	4.87E+03	3.66E+04	1.18E+05	5.50E+03	5.56E+04
	2	2.19E+05	5.01E+03	3.45E+04	1.09E+05	5.26E+03	5.06E+04
**λ_{ex} = 420 nm**	0	4.15E+04	1.52E+03	4.77E+03	5.28E+03	1.36E+03	2.25E+03
	0.1	3.28E+04	1.36E+03	5.73E+03	1.18E+04	1.46E+03	5.74E+03
	0.2	2.81E+04	1.21E+03	6.71E+03	1.70E+04	1.57E+03	8.33E+03
	0.3	2.38E+04	1.15E+03	7.43E+03	2.13E+04	1.80E+03	1.09E+04
	0.4	2.18E+04	1.11E+03	7.51E+03	2.27E+04	1.93E+03	1.19E+04
	0.5	1.88E+04	1.02E+03	8.17E+03	2.67E+04	2.21E+03	1.45E+04
	0.6	1.69E+04	9.04E+02	7.59E+03	2.50E+04	2.30E+03	1.45E+04
	0.7	1.43E+04	8.82E+02	7.63E+03	2.57E+04	2.67E+03	1.65E+04
	0.8	1.24E+04	7.96E+02	7.66E+03	2.68E+04	2.34E+03	1.59E+04
	0.9	1.33E+04	9.10E+02	9.47E+03	3.36E+04	2.44E+03	1.91E+04
	1	1.36E+04	8.39E+02	8.41E+03	3.06E+04	2.52E+03	1.83E+04
	1.1	1.69E+04	9.34E+02	9.66E+03	3.49E+04	2.57E+03	2.01E+04
	1.2	2.29E+04	1.14E+03	1.07E+04	3.64E+04	2.73E+03	2.08E+04
	1.4	4.97E+04	1.64E+03	1.27E+04	4.27E+04	2.94E+03	2.33E+04
	1.6	6.74E+04	1.64E+03	1.11E+04	3.45E+04	2.50E+03	1.84E+04
	1.8	8.15E+04	1.94E+03	1.23E+04	3.75E+04	2.62E+03	1.99E+04
	2	8.36E+04	1.97E+03	1.26E+04	3.78E+04	2.81E+03	2.08E+04

Wavelength range for selected emission bands are as follows, with colors corresponding to figure below. UO_2 emission; 500-574 nm (green). Eu(III) ΔJ = 0; 578-581 nm (purple). Eu(III) ΔJ = 1; 585-598 nm (blue). Eu(III) ΔJ = 2; 608-627 nm (red). Eu(III) ΔJ = 3; 647-657 nm (orange). Eu(III) ΔJ = 4; 681-711 nm (yellow).

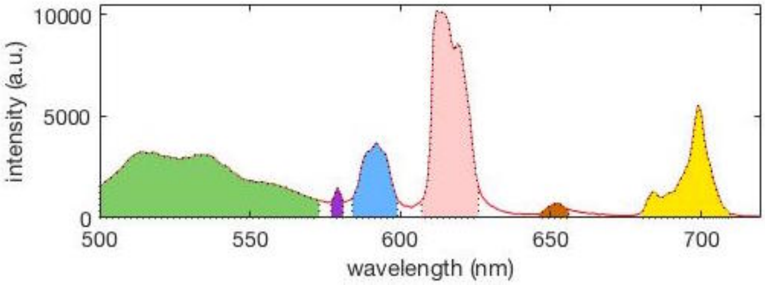

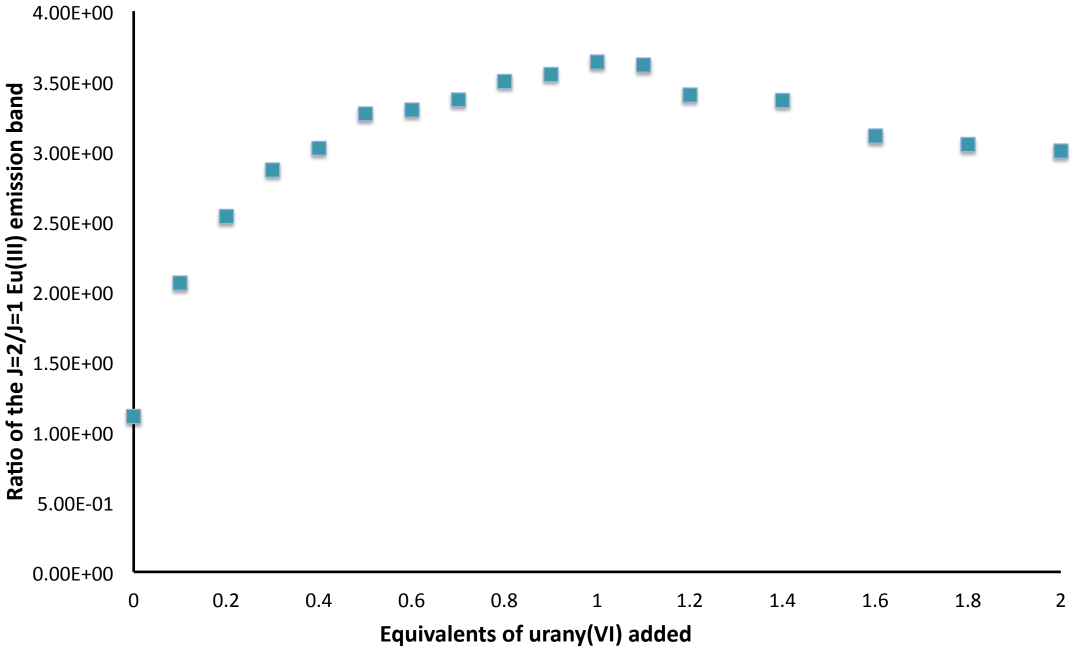

Figure S8. Plot of the Eu(III) $^5D_0 \rightarrow {}^7F_2$ to $^5D_0 \rightarrow {}^7F_2$ integrated emission ratio ($\Delta J=2/\Delta J=1$) against the number of equivalents of uranyl(VI) added. Typically, the ratio of these bands gives an indication of the site symmetry around the Eu(III) ion, with a ratio >8 indicating that Eu(III) lies in a low symmetry environment and a ratio of <0.7 suggesting the Eu(III) ion lies in a centrosymmetric coordination environment.[12]

References

(1) (a) Abada, S.; Lecointre, A.; Elhabiri, M.; Esteban-Gómez, D.; Platas-Iglesias, C.; Tallec, G.; Mazzanti, M.; Charbonnière, L. J. *Chem. Commun.* **2012**, *48*, 4085. (b) Elhabiri, M.; Abada, S.; Sy, M.; Nonat, A.; Choquet, P.; Esteban-Gómez, D.; Cassino, C.; Platas-Iglesias, C.; Botta, M.; Charbonnière, L. J. *Chem. Eur. J.* **2015**, *21*, 6535.

(2) (a) Gampp, H. ;Maeder, M. ; Meyer, C. J.; Zuberbuhler, A. D. *Talanta*, **1985**, *32*, 1133. (b) Gampp, H. ;Maeder, M. ; Meyer, C. J.; Zuberbuhler, A. D. *Talanta*, **1986**, *33*, 943.

(3) a) H. Gampp, M. Maeder, C. J. Meyer and A. D. Zuberbühler, Talanta, **1985**, *32*, 95. b) H. Gampp, M. Maeder, C. J. Meyer and A. D. Zuberbühler, Talanta, **1985**, *32*, 257.

(4) Tao, J. M.; Perdew, J. P.; Staroverov, V. N.; Scuseria, G. E. *Phys. Rev. Lett.* **2003**, *91*, 146401.

(5) Gaussian 09, Revision D.01, Frisch, M. J.; Trucks, G. W.; Schlegel, H. B.; Scuseria, G. E.; Robb, M. A.; Cheeseman, J. R.; Scalmani, G.; Barone, V.; Mennucci, B.;

Petersson, G. A.; Nakatsuji, H.;Caricato, M.; Li, X.; Hratchian, H. P.; Izmaylov, A. F.; Bloino, J.; Zheng, G.; Sonnenberg, J. L.; Hada, M.; Ehara, M.; Toyota, K.; Fukuda, R.; Hasegawa, J.; Ishida, M.; Nakajima, T.; Honda, Y.; Kitao, O.; Nakai, H.; Vreven, T.; Montgomery, Jr., J. A.; Peralta, J. E.; Ogliaro, F.; Bearpark, M.; Heyd, J. J.; Brothers, E.; Kudin, K. N.; Staroverov, V. N.; Kobayashi, R.; Normand, J.; Raghavachari, K.; Rendell, A.; Burant, J. C.; Iyengar, S. S.; Tomasi, J.; Cossi, M.; Rega, N.; Millam, N. J.; Klene, M.; Knox, J. E.; Cross, J. B.; Bakken, V.; Adamo, C.; Jaramillo, J.; Gomperts, R.; Stratmann, R. E.; Yazyev, O.; Austin, A. J.; Cammi, R.; Pomelli, C.; Ochterski, J. W.; Martin, R. L.; Morokuma, K.; Zakrzewski, V. G.; Voth, G. A.; Salvador, P.; Dannenberg, J. J.; Dapprich, S.; Daniels, A. D.; Farkas, Ö.; Foresman, J. B.; Ortiz, J. V.; Cioslowski, J.; Fox, D. J. Gaussian, Inc., Wallingford CT, 2009.

(6) Dolg, M.; Stoll, H.; Savin, A.; Preuss, H. Energy-adjusted Pseudopotentials for the Rare Earth Elements.*Theor.Chim. Acta.* **1989**, *75*, 173.

(7) A. Moritz, M. Dolg, Theor. Chem. Acc. **2008**, *121*, 297.

(8) Tomasi, J.; Mennucci, B.; Cammi, R. Quantum Mechanical Continuum Solvation Models.*Chem. Rev* .**2005**, *105*, 2999.

(9) Rappe, A. K.; Casewit, C. J.; Colwell, K. S.; Goddard,W. A., III; Skiff, W. M. UFF, a Full Periodic Table Force Field for Molecular Mechanics and Molecular Dynamics Simulations. *J. Am. Chem. Soc.* **1992**, *114*, 10024.

(10) (a) Mato-Iglesias, M.; Balogh, E.; Platas-Iglesias, C.; Toth, E.; de Blas, A.; Rodriguez Blas, T. *Dalton Trans.* **2006**, 5404. (b) Kotek, J.; Lebduskova, P; Hermann, P.; Vander Elst, L.; Muller, R. N.; Geraldes, C. F. G. C.; Maschmeyer, T.; Lukes, I.; Peters, J. A. *Chem. Eur. J.* **2003**, *9*, 5899. (c) Sherry, A. D.; Ren, J.; Huskens, J.; Brucher, E.; Toth, E.; Geraldes, C. F. G. C.; Castro, M.M. C. A.; Chacheris, W. P. *Inorg. Chem.* **1996**, *35*, 4604.

(11) Nchimi Nono, K.; Lecointre, A.; Regueiro-Figueroa, M.; Platas-Iglesias, C.; Charbonnière, L.J. *Inorg. Chem.* **2011**, *50*, 1659.

(12) (a) Richardson, F.S.; *Chem. Rev.* **1982**, *82*, 541. (b) Tedeschi, C.; Azema, J.; Gornitzka, H.; Tisnes, P.; Picard C. *Dalton. Trans.* **2003**, 1738. (c) Brito, H.F.; Malta, O.L.; Menezes, J.F.S. *J. Alloys Compd*. **2000**, *303*, 336.
